# The Modification of a Tetrafunctional Epoxy and Its Curing Reaction

**DOI:** 10.3390/ma8063671

**Published:** 2015-06-18

**Authors:** Mingming Yu, Bin Feng, Wang Xie, Lin Fang, Hong Li, Liqi Liu, Musu Ren, Jinliang Sun, Jiabao Zhang, Hefeng Hu

**Affiliations:** Research Center for Composite Materials, Shanghai University, Shanghai 200072, China; E-Mails: fengbing@shu.edu.cn (B.F.); wangxie@shu.edu.cn (W.X.); fanglin7@126.com (L.F.); lihong2007@shu.edu.cn (H.L.); llq@shu.edu.cn (L.L.); msren@shu.edu.cn (M.R.); jlsun@shu.edu.cn (J.S.); jbzhang@shu.edu.cn (J.Z.); huhefeng@shu.edu.cn (H.H.)

**Keywords:** modification, toughen, TGDDE, dimer fatty acid (DFA), curing reaction

## Abstract

Recent experimental results showed that the *T*_g_ of cured resin scarcely decreased and the impact strength of resins increased by over 50% when a tetrafunctional epoxy named *N*,*N*,*N*',*N*'-tetraglycidyl-4,4'-diaminodiphenyl ether (TGDDE) was introduced to an appropriate flexible chain from a dimer fatty acid (DFA). In order to understand the reason for this phenomenon, the modification and the chemical structure of the prepolymer together with the curing reaction and the viscoelasticity of the cured resins were studied in detail in the present work. The results indicated that the modification would help the prepolymer improve its molecular mobility. As a result, the resins could be further cured, resulting in the cross-linking density increasing. This is because the curing efficiency was increased, but the tetrafunctional epoxy was not cured completely due to its large steric hindrance. Moreover, the flexibility of some parts of the networks was improved, which was beneficial for the toughness of the cured resins. Therefore, the toughness of the tetrafunctional resin was improved with little influence on the thermal properties when the epoxies were modified with an appropriate content of DFA.

## 1. Introduction

**Scheme 1:**
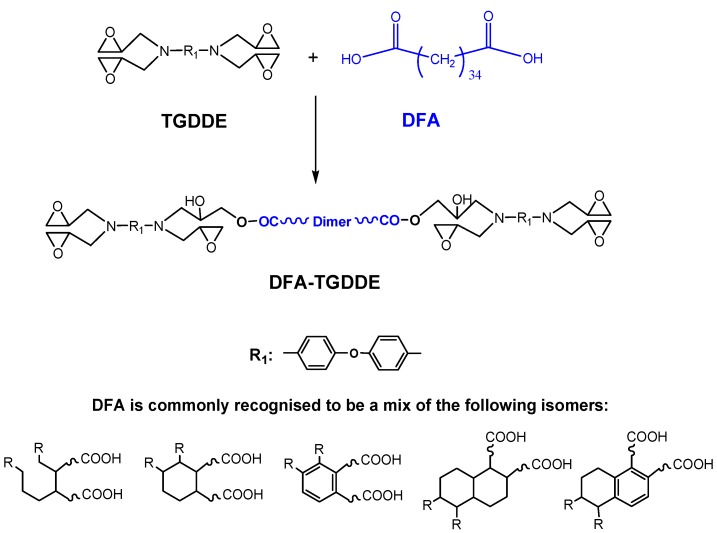
The reaction between TGDDE and dimer fatty acid (DFA).

**Scheme 2:**
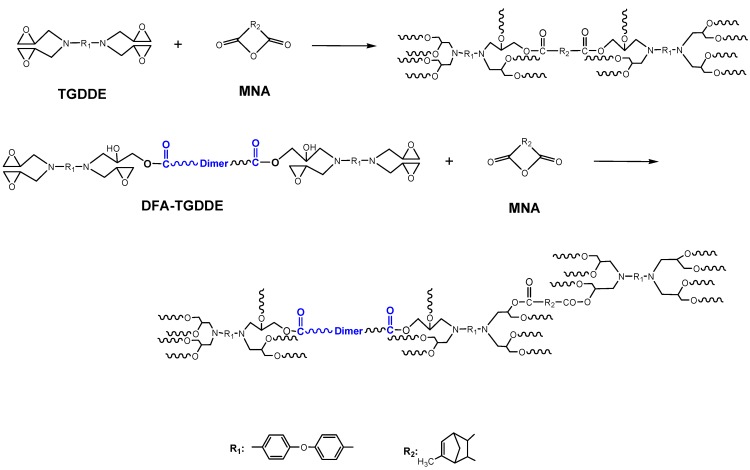
The curing reaction of TGDDE/methyl nadic anhydride (MNA) and DFA-TGDDE/MNA systems.

**Scheme 3:**
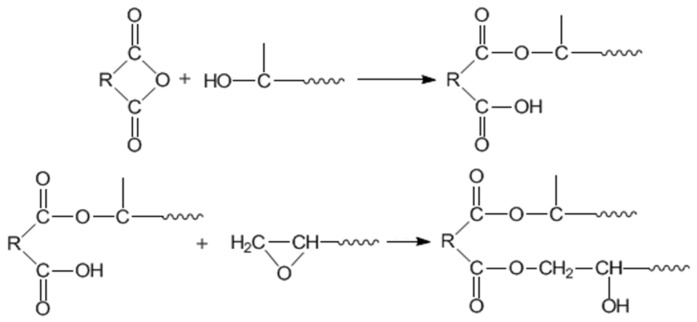
The curing reaction between epoxy and anhydride (absence of catalyst).

As important thermosetting epoxy polymers, the amine based tetrafunctional epoxies were widely used as the matrices for fiber-composites materials [1,2,3,4]. Among these epoxies, *N*,*N*,*N*',*N*'-tetraglycidyl-4,4'-diaminodiphenyl ether (TGDDE) was deemed the most commonly used one, because it had low viscosity, high reactivity and excellent thermal properties. However, the pure cured resins tend to be too brittle to be applied. Many methods were used to improve the toughness of the resin, including the modification on the molecular main chain by introducing a flexible chain, organic/inorganic hybridization, nanoparticle reinforcement, high temperature thermo-plastic blending toughening and aggregation state optimization [5,6,7,8,9]. Among these, the molecular chain modification with a flexible chain was an efficient way for improving the toughness of epoxy resins. However, it was nearly selected as a priority toughening method for the tetrafunctional epoxy resins, because this method was regarded to have a dispositive effect on the thermal property of the cured resin. However, the recent experiment results indicated that *T*_g_ of the cured resin scarcely decreased and the impact strength of the resins increased by over 50% when TGDDE was modified with an appropriate dimer fatty acid (DFA) content [10], which verified that the tetrafunctional epoxy resin could be toughened by introducing a befitting flexible chain with the premise of little influence on the thermal properties. In order to understand the reason for this phenomenon, in the present work, TGDDE was modified with DFA as in Scheme 1. The modification was studied by Differential Scanning Calorimetry (DSC) method and Fourier Transform Infrared Spectroscopy (FTIR) dynamic method. Meanwhile, the chemical structure of the prepolymer was characterized by FTIR. After that, the epoxies were cured with methyl nadic anhydride (MNA) as Scheme 2 and Scheme 3, respectively. The curing behavior and curing kinetics of TGDDE/MNA and DFA-TGDDE/MNA systems were investigated by a non-isothermal DSC method. Moreover, the viscoelasticity of the cured resins was studied by the thermomechanical properties from dynamic mechanical analysis (DMA).

## 2. Results and Discussion

### 2.1. Modification of TGDDE with DFA

The modification of TGDDE with DFA was through the reaction between the epoxy groups from TGDDE and the carboxyl groups from DFA, as Scheme 1 shows. The reaction process was characterized with the DSC method and the dynamic FTIR method. 

**Figure 1:**
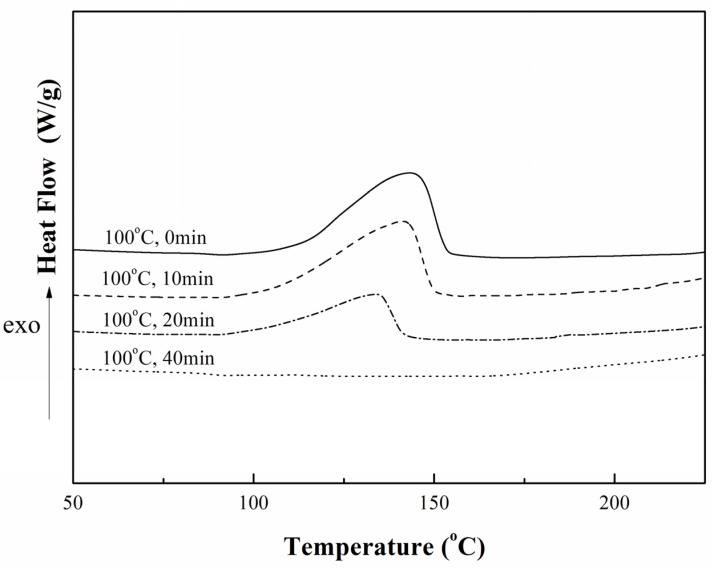
Differential Scanning Calorimetry (DSC) curves of 6%DFA-TGDDE systems at various reaction times, 10 °C/min.

The thermal behaviors of the reactions were studied by the DSC method, as shown in Figure 1. It can be seen that the exothermic reaction took place slowly and reached the maximum reaction rate at the peak temperature of ~143 °C which increased slightly with the reaction proceeding, and then the reaction slowed down because less unreacted material was available. Moreover, the results revealed that the reaction enthalpy disappeared when the modification carried out under 100 °C for 40 min, which illustrated that the reaction was accomplished at that condition.

**Figure 2:**
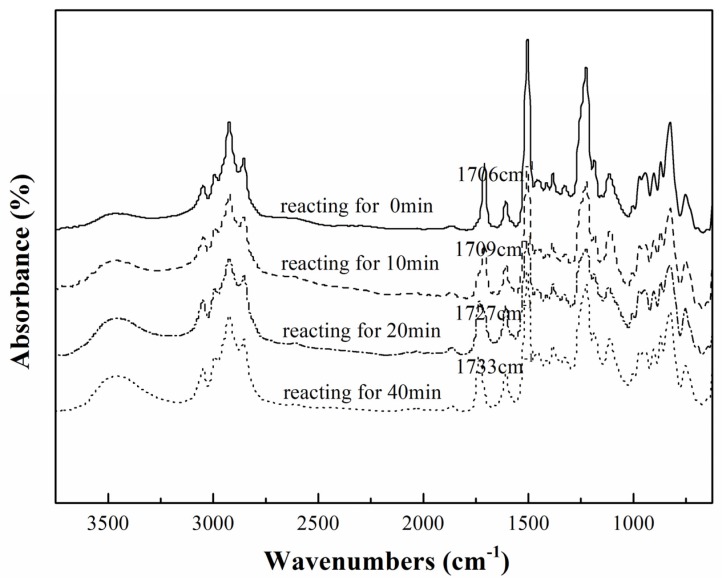
The chemical shifts of the modification process of 6%DFA-TGDDE systems.

In addition, as shown in Figure 2, the dynamic FTIR results indicated that the spectrum of carbonyl (1706–1733 cm^−1^) was shifted up with the reaction proceeding, which indicated that the ester group was being formed with the occurrence of the reaction between the carboxyl group and the epoxy group. 

These results illustrated that the modification was accomplished under 100 °C for 40 min, and the flexible chain from DFA was introduced into the molecular chain of the epoxy. 

**Figure 3:**
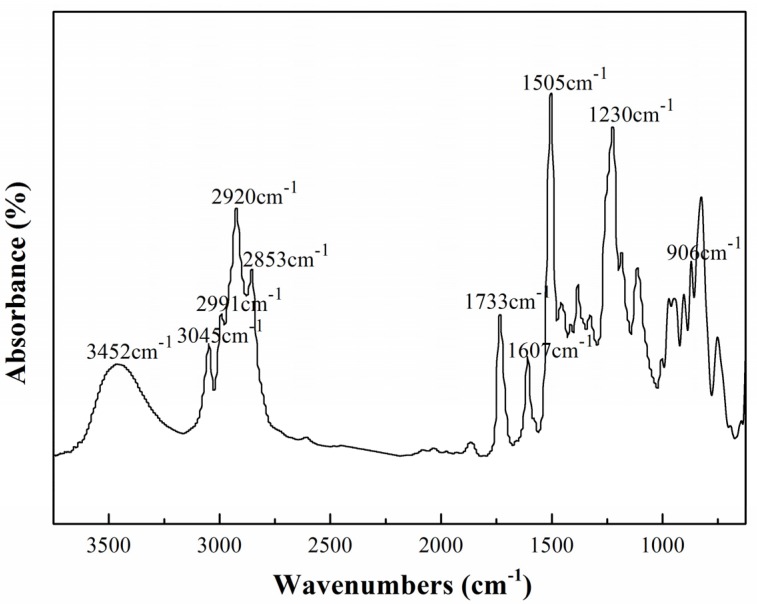
FTIR spectra of the 6%DFA-TGDDE systems.

Furthermore, the chemical structure of the modified epoxy (DFA-TGDDE) was characterized by FTIR, as Figure 3 shown. The spectra were assigned as follows: 3452 cm^−1^ (O–H), 3045 cm^−1^, 2991 cm^−1^, 2920 cm^−1^ and 2853 cm^−1^ (C–H), 1733 cm^−1^ (C=O), 1607 cm^−1^ and 1505 cm^−1^ (phenylene), 1230 cm^−1^ (Ar–O–Ar), 906 cm^−1^ (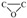). 

### 2.2. Curing Reactions of TGDDE/MNA and DFA-TGDDE/MNA Systems

In order to investigate the influence of the modification on the curing reactions, the curing behaviors and the curing kinetics of TGDDE/MNA and DFA-TGDDE/MNA were studied in detail. 

#### 2.2.1. Curing Behaviors

**Figure 4:**
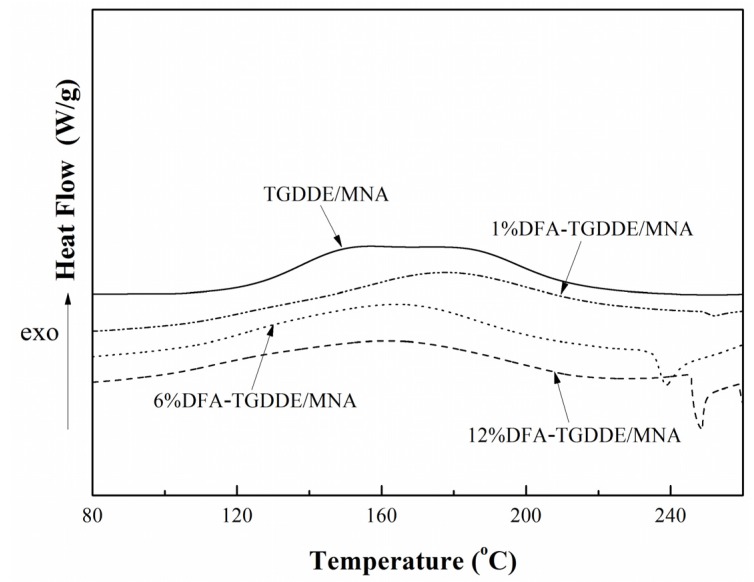
DSC curves of TGDDE and DFA-TGDDE curing with methyl nadic anhydride (MNA), 5 °C/min.

The curing behaviors of TGDDE/MNA and DFA-TGDDE/MNA systems were shown in Figure 4. The initial curing temperature (*T*_i_), the peak curing temperature (*T*_p_), the finishing temperature (*T*_f_), and the curing reaction enthalpy (Δ*H*) of these curing reactions were summarized in Table 1. Besides, the curing reaction between epoxy and anhydride occurred as in Scheme 3, which showed that the reaction occurred with two reactions and the reaction rate was controlled by the content of hydroxyl groups when the curing system was without catalyst. Therefore, the curing curve of TGDDE/MNA system showed bimodal character and the curves of DFA-TGDDE/MNA exhibited unimodal character, which were ascribed to the acceleration caused by the increasing content of hydroxyl groups. In addition, the results showed that the curing efficiency could be observably improved when the tetrafunctional epoxy modified with DFA since the initial curing temperature decreased with the increasing content of DFA. This phenomenon was also caused by the increasing content of hydroxyl groups. Additionally, the curing reaction enthalpy of all the systems pointed out that the curing system was further reacted after the modification. It is generally known that the multifunctional epoxy/anhydride systems were too difficult to be cured fully due to their large steric hindrance. However, the modification could improve the post curing reactions since the aliphatic chain of DFA would improve the mobility of the cross-linking networks in the latter curing reaction stage. Therefore, the curing systems were further cured when the epoxy was modified with DFA.

All the results indicated that the curing reactions could be improved when the tetrafunctional epoxy was modified with DFA.

**Table 1:** Characteristics of the curing systems, 5 °C/min.

Systems	*T*_i_ (°C)	*T*_p_ (°C)	*T*_f_ (°C)	Δ*H* (J·g^−1^)
TGDDE/MNA	122	187	216	109.5
1%DFA-TGDDE/MNA	105	177	219	155.0
6%DFA-TGDDE/MNA	98	165	213	189.3
12%DFA-TGDDE/MNA	89	162	213	199.4

#### 2.2.2. Curing Kinetics

**Figure 5:**
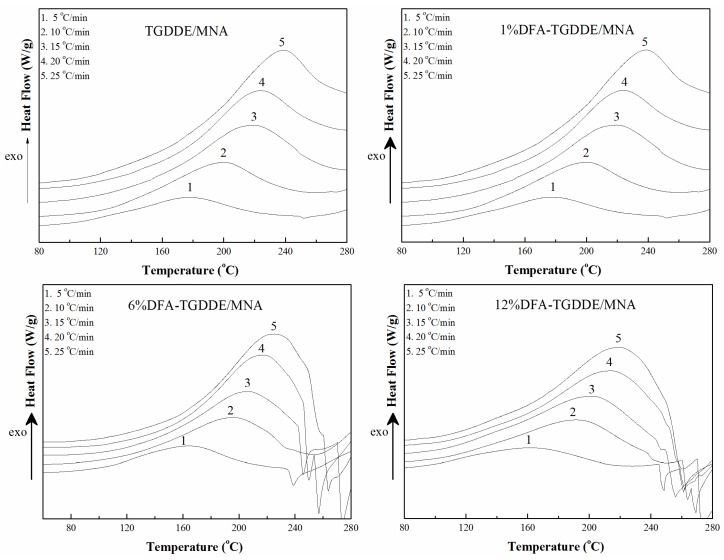
Enthalpy *vs.* Temperature of the curing process at different curing rate.

**Figure 6:**
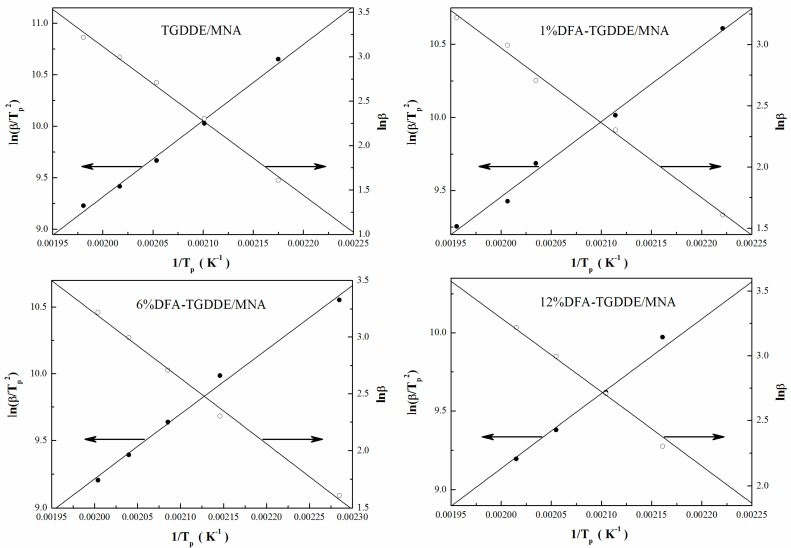
ln(β*/T*^2^*_p_*) and lnβ *vs.* 1/*T*_p_ of the curing systems.

In order to further investigate the influence of the modification on the curing reactions, the reaction parameters of TGDDE/MNA and DFA-TGDDE/MNA systems were calculated from the curing kinetics with a non-isothermal DSC method [11], and the DSC curves of the curing reactions with different heating rates were shown in Figure 5. As a reactivity parameter, the apparent activation energies (*E_a_*) were calculated with the average values of the activation energy simulated by Kissinger’s equation (*E_k_*) as Equation (1) [12] and the activation energy simulated by Ozawa’s equation (*E_o_*) as in Equation (2) [13]. The results were listed in Table 2. It is shown that the curing efficiency increased after the modification since the apparent activation energies (*E_a_*) decreased with the increasing content of DFA:

(1) $$\ln(\frac{\beta }{T_{p}^{2}})=-\frac{E_{k}}{R}\frac{1}{T_{p}}\text{+}\ln\frac{AR}{E_{k}}$$

(2) $$E_{o}=-\frac{R}{1.052}\frac{\text{d}\ln\beta }{\text{d}(1/T_{p})}$$

where *E_k_* is the curing activation energy simulated by Kissinger’s equation, *E_o_* is the curing activation energy simulated by Ozawa’s equation. β is the heating rate and *T_p_* is the maximum peak temperature, and *R* is the ideal gas constant. The value of *E_k_* can be determined with a plot of ln*(*β/*T*^2^*_p_) vs.* 1/*T_p_* as shown in Figure 6.

Additionally, as a steric hindrance parameter of the prepolymer, the pre-exponential factor (*A*) was calculated as Equation (3), and the results were listed in Table 2. The results showed that the *A* values sharply decreased after the modification, which illustrated that the modification would improve the flexibility of the molecular chain:

(3) $$A\approx \frac{\beta E\exp(E/RT_{p})}{R{T_{P}}^{2}}$$

**Figure 7:**
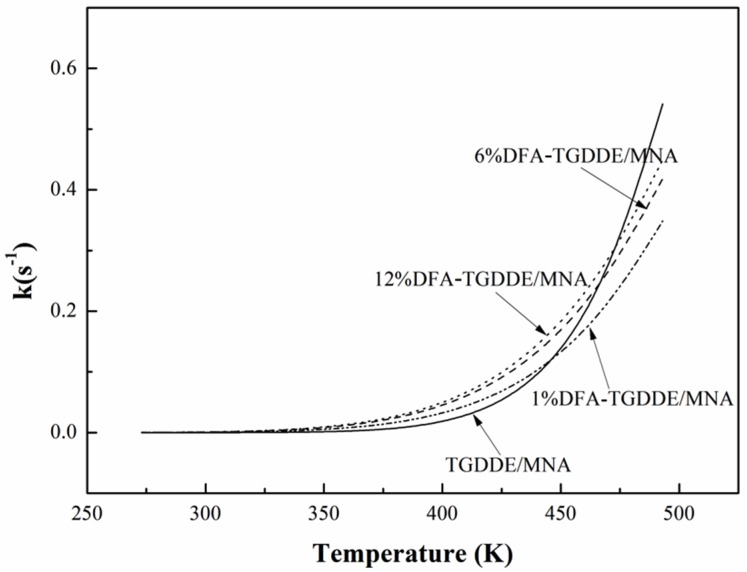
Curing reaction rate constant (*k*) values at different temperatures.

Furthermore, the reaction rate constant (*k*) was calculated from an Arrhenius equation (as Equation (4)). The *k* values at different temperatures were shown in Figure 7. The value of *k* was dependent on the temperature and increased with an increasing curing temperature. Moreover, when the curing temperature was below 160 °C (433 K), the values of *k* increased in the following order: TGDDE/MNA < 1%DFA-TGDDE/MNA < 6%DFA-TGDDE/MNA < 12%DFA-TGDDE/MNA. This revealed that the curing reaction rate was increased at the initial reaction stage when TGDDE was modified with DFA, and the influence would be much more remarkable with the increasing content of DFA:

(4) k=Aexp(−E/RT)

In addition, the reaction order *n* was obtained from the Crane’s equation (as Equation (5)) [14], and the results were listed in Table 2. The calculated *n* values of these curing reactions were all close to 0.9, which illustrated that all these reactions could be approximately similar to the first order reaction:

(5) $$\frac{1}{n}=\frac{R}{E}\frac{\text{d}(\ln\beta )}{\text{d}(1/T_{p})}$$

**Table 2:** The curing kinetic parameters of the curing systems.

Systems	*E_k_* (kJ/mol)	*E_o_* (kJ/mol)	*E* (kJ/mol)	*A* (s^−1^)	*n*
TGDDE/MNA	61.57	66.13	63.85	1.81 × 10^6^	0.918
1%DFA-TGDDE/MNA	42.88	48.32	45.60	1.22 × 10^4^	0.897
6%DFA-TGDDE/MNA	40.09	45.47	42.78	7.39 × 10^3^	0.894
12%DFA-TGDDE/MNA	39.65	45.01	42.33	7.14 × 10^3^	0.894

From the four calculated curing reaction parameters, it can be seen that the curing efficiency and the initial curing reaction rate would be increased on account of the decreasing activation energy and the more flexible molecular chain when TGDDE was modified with DFA. However, the reaction order *n* was not affected after the modification, and all these reactions could be approximately similar to the first order reaction.

### 2.3. Viscoelasticity of the Cured Resins

**Figure 8:**
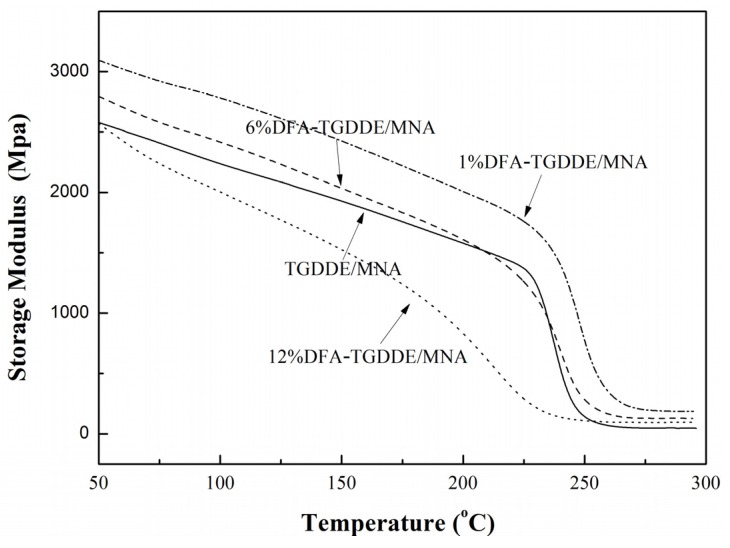
Storage modulus curves of the cured resins, 5 °C/min, 1 Hz.

**Figure 9:**
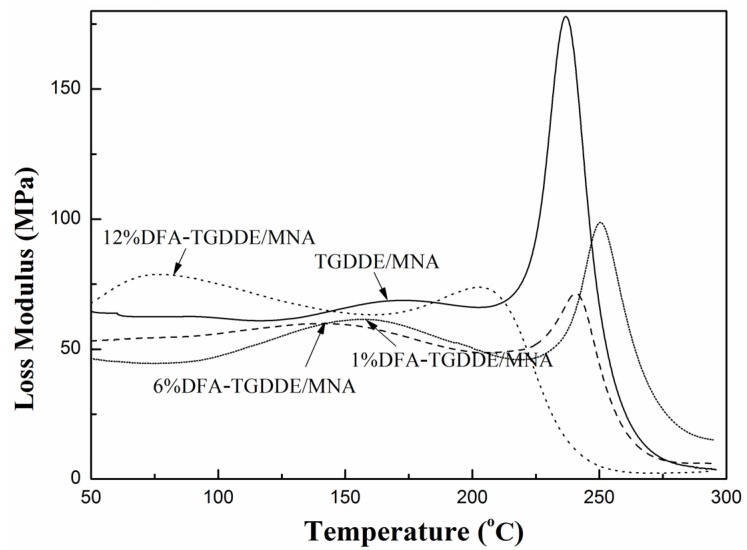
Loss modulus curves of the cured resins, 5 °C/min, 1 Hz.

**Figure 10:**
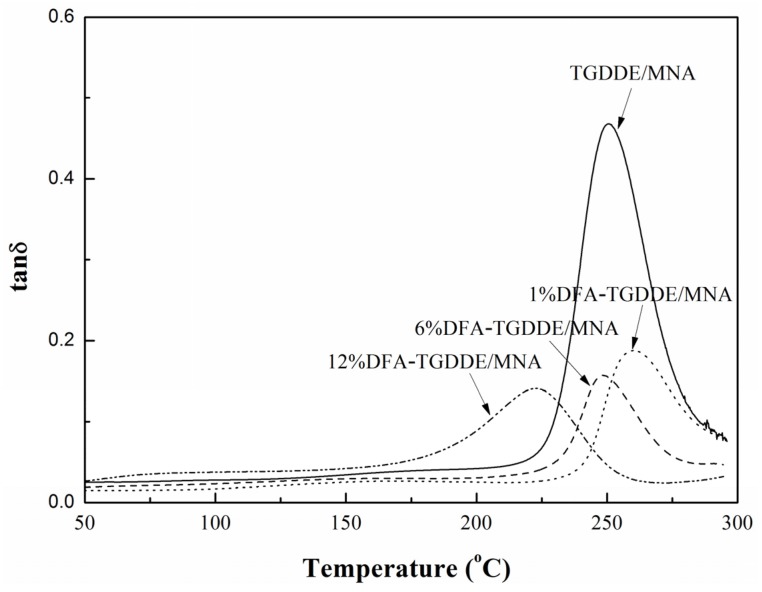
Tanδ curves of the cured resins, 5 °C/min, 1 Hz.

The viscoelasticity of the cured resins was studied with their dynamic mechanical properties. As Figure 8 shows, the storage modulus of the cured resins decreased with increasing temperature due to the increasing flexibility of the molecular chain. Moreover, the modulus could be increased when the epoxy modified with an appropriate content DFA. Besides, the glass transition temperature (*T*_g_) of the cured resin was calculated based on the rubber elasticity theory, and results were listed in Table 3. The results showed that the *T*_g_ of the cured resin was not obviously affected when the epoxy was modified with an appropriate content DFA. On the other hand, Figure 9 showed that the peak-intensities near the *T*_g_ of the loss modulus curves of DFA modified resin exhibited obvious reduction and the peak-width of them widened, which indicates that the stiffness of networks was relaxed because a flexible chain was introduced to the networks when the epoxy was modified with DFA. The effect was more obvious with increasing DFA content. In addition, the curves showed that the modified resins had obvious secondary relaxation at lower temperature, which was also caused by the mobility of a flexible chain of the networks. Additionally, Figure 10 showed tanδ (tanδ = *E*''/*E*') curves of the cured resin, it illustrated that all the crosslinking networks were homogeneous systems since the curves exhibited one peak. Moreover, the curves indicated that the crosslinking density was related to the modifier, and it was not markedly affected when the epoxy was modified with an appropriate content DFA.

All the results illustrated that the toughness of the cured resins was improved after the modification, and the effect was more obvious with the DFA content. In addition, it can be seen that the *T*_g_ values and the cross-linking density of the cured resins were not remarkably affected when TGDDE was modified with an appropriate content DFA. However, the excess aliphatic chain would decrease the cross-linking density of the cured resins. 

**Table 3:** The *T*_g_ and impact strength of the cured resins.

Systems	DFA (g)	TGDDE (g)	*T*_g_ (°C)	Impact Strength (kJ/m^2^)
TGDDE/MNA	–	100	230	4.14
1%DFA-TGDDE/MNA	2	100	237	5.59
6%DFA-TGDDE/MNA	14	100	228	6.31
12%DFA-TGDDE/MNA	30	100	188	7.13

## 3. Experimental Section 

### 3.1. Materials

Methyl nadic anhydride (MNA) was purchased from Aldrich (St. Louis, MO, USA). Dimer acid (DFA, PRIPOL 1013) with acid value 195 mg/KOH was obtained from Croda (Cowick Hall, UK). *N*,*N*,*N*',*N*'-tetraglycidyl-4,4'-diaminodiphenyl ether (TGDDE) with epoxy equivalent weight (EEW) 120 g/mol was supplied by department of polymer materials of Shanghai University. All chemical agents were used without further purification.

### 3.2. Preparation of DFA-TGDDE

In order to investigate the modification between TGDDE and DFA, these two compounds were mixed at room temperature, and then reacted at 100 °C for several minutes. Thus, four samples were prepared, which were the system of TGDDE mixing with DFA at room temperature (assuming unreacted, named reacting for 0 min), the system of TGDDE reacting with DFA for 10 min at 100 °C (named reacting for 10 min), the system of TGDDE reacting with DFA for 20 min at 100 °C (named reacting for 20 min) and the system of TGDDE reacting with DFA for 40 min at 100 °C (named reacting for 40 min).

Additionally, DFA-TGDDE was prepared as the formula showed in Table 4, and the mixture was reacted in a three necked flask with stirring at 100 °C for 40 min.

**Table 4:** The formula of DFA-TGDDE and their epoxy equivalent weight (EEW).

Systems	DFA (g)	TGDDE (g)	EEW (g/mol)
1%DFA-TGDDE	2	100	121
6%DFA-TGDDE	14	100	128
12%DFA-TGDDE	30	100	137

### 3.3. Preparation of Cured Epoxy Resins

The modified epoxy was mixed with a stoichiometric amount of the curing agent (MNA) as Table 5 shown, and then the mixture was heated to 65 °C under vacuum to remove air bubbles and moisture. Subsequently, the mixture was cured at 100 °C for 2 h, followed by curing at 160 °C for 2 h, and finally post cured at 200 °C for 4 h.

**Table 5:** The formula of TGDDE/MNA and DFA-TGDDE/MNA systems.

Systems	Epoxy (g)	Curing Agent (g)
TGDDE/MNA	100	126
1%DFA-TGDDE/MNA	100	125
6%DFA-TGDDE/MNA	100	118
12%DFA-TGDDE/MNA	100	110

### 3.4. Characterization

FTIR spectrum was recorded on a Nicolet 380 infrared spectrometer (Waltham, MA, USA) in the range of 4000–400 cm^−1^, and the sample was spread on the KBr slice.

Differential Scanning Calorimetry (DSC) was performed on a TA Q2000 (New Castle, MO, USA) with a constant nitrogen flow of 50 mL/min. About 5 mg of a sample (DFA-TGDDE, TGDDE/MNA, DFA-TGDDE/MNA) was weighted and put into a hermetic aluminum sample pan at 25 °C, which was then sealed, and the sample was tested immediately. The dynamic scanning experiment ranged from 80 to 270 °C. The curing behaviors and the curing kinetics were studied with a non-isothermal DSC method.

Dynamic mechanical analysis (DMA) was characterized with a TA Q800 in the air. The specimen of 60 mm × 10 mm × 3 mm was loaded in a three-point bending mode from 50 to 300 °C at a heating rate of 5 °C/min with a frequency of 1 Hz.

## 4. Conclusions

A tetrafunctional epoxy named TGDDE was modified with DFA, and the prepolymer was cured with MNA. The modification and curing reaction together with the viscoelasticity of the cured resins were studied in detail. The results from the modification verified that the flexible chain from DFA was introduced into the molecular chain of the epoxies when TGDDE reacted with DFA under 100 °C for 40 min. Moreover, the curing reaction parameters revealed that the prepolymer could be further cured when TGDDE was modified with DFA. This is because that the curing efficiency was improved on account of the decreasing activation energy and the improving molecular chain mobility, while the tetrafunctional epoxy was hardly cured completely duo to its large steric hindrance. Thus, the cross-linking density of the networks was not remarkably influenced when TGDDE was modified with an appropriate content of DFA, although the long flexible chain would decrease the cross-linking density. On the other hand, the viscoelasticity of the cured resins illustrated that the modification would improve the mobility of the networks, which was beneficial for the toughness of the cured resin. Therefore, the toughness of the tetrafunctional resin was improved with little influence on the thermal properties when the epoxies were modified with an appropriate content of DFA.
